# Flexibility Services Based on OpenADR Protocol for DSO Level

**DOI:** 10.3390/s20216266

**Published:** 2020-11-03

**Authors:** Juan Ignacio Guerrero Alonso, Enrique Personal, Sebastián García, Antonio Parejo, Mansueto Rossi, Antonio García, Federico Delfino, Ricardo Pérez, Carlos León

**Affiliations:** 1 Department of Electronic Technology, ETSII, Universidad de Sevilla, 41012 Sevilla, Spain; 2 Department of Electronic Technology, EPS, Universidad de Sevilla, 41011 Sevilla, Spain; epersonal@us.es (E.P.); sgarcia15@us.es (S.G.); aparejo@us.es (A.P.); antgar@us.es (A.G.); cleon@us.es (C.L.); 3 DITEN—Electrical, Electronics and Telecommunication Engineering and Naval Architecture Department, Savona Campus, University of Genoa, 16145 Genoa, Italy; mansueto.rossi@unige.it (M.R.); federico.delfino@unige.it (F.D.); 4 Business Unit Manager at Enel-Endesa, 00198 Rome, Italy; ricardo.perezs@enel.com

**Keywords:** smart grid, demand-side management, flexibility market, OpenADR standard, Blockchain

## Abstract

Nowadays, Distribution System Operators are increasing the digitalization of their smart grids, making it possible to measure and manage their state at any time. However, with the massive eruption of change-distributed generation (e.g., renewable resources, electric vehicles), the grid operation have become more complex, requiring specific technologies to balance it. In this sense, the demand-side management is one of its techniques; the demand response is a promising approach for providing Flexibility Services (FSs) and complying with the regulatory directives of the energy market. As a solution, this paper proposes the use of the OpenADR (Open Automated Demand Response) standard protocol in combination with a Decentralized Permissioned Market Place (DPMP) based on Blockchain. On one hand, OpenADR hierarchical architecture based on distributed nodes provides communication between stakeholders, adding monitoring and management services. Further, this architecture is compatible with an aggregator schema that guarantees the compliance with the strictest regulatory framework (i.e., European market). On the other hand, DPMP is included at different levels of this architecture, providing a global solution to Flexibility Service Providers (FSP) that can be adapted depending on the regulation of a specific country. As a proof of concept, this paper shows the result of a real experimental case, which implements a Capacity Bidding Program where the OpenADR protocol is used as a communication method to control and monitor energy consumption. In parallel, the proposed DPMP based on Blockchain makes it possible to manage the incentives of FSs, enabling the integration of local and global markets.

## 1. Introduction

The target of Distribution System Operators (DSOs) is to operate and manage distribution networks in a safe and secure manner. They are also responsible for developing, managing, and maintaining the distribution grids to ensure the long-term ability of the system to deliver high quality services to grid consumers and other stakeholders of the electric power grid. In this sense, the European DSOs have different concerns [1]:

- In European countries, the DSOs are not allowed to own batteries or any other element involved in the energy markets [2,3]. However, the Latin American market [4] is quite different, and the regulation differs from the European market, providing opportunities for testing new technologies and their advantages in the DSO power grid. Additionally, the regulation varies for each country. Thus, a general solution independent from the regulation is necessary.
- The deployment of Distributed Energy Resources (DER), mainly based on renewable energy generation, at different voltage levels, is changing the traditional distribution schema [5].
- The new horizon of the mobility solution, with the deployment of millions of electric vehicles and necessary recharging infrastructure.
- The Voltage and Reactive Power (Volt-VAR) control and the congestion management in the new scenario created by smart grids.
- The need to improve the ability of current Supervisory Control and Data Acquisition (SCADA) to support the new Intelligent Electronic Devices (IEDs).

These concerns provoke several problems for the DSO [6], which has more difficulties maintaining the power grid reliability and stability, making very difficult the integration of DERs in the power grid, at medium and low voltages. Thus, in this case, the DSO needs a global solution, which could be applied in any place, including Europe. In the European Union, there are special cases, like the case of Germany, in which there is specific regulation [7] about the connection of renewable resources into low voltage, which provides additional advantages in Flexibility Services (FSs). Flexibility is the capacity to adjust loads, power generation and storage in real time, at the level of clients, including the medium and low voltage clients, adapting their behaviour to the power grid needs at each moment. Thus, the FSs are provided by aggregators, asset owners, and others in performing optimizations based on DER management, and these actors are usually named Flexibility Service Providers (FSP). When the DSO performs the Flexibility Request; FPSs are addressed to increase power quality and grid stability, decreasing grid congestion. At the same time, the optimization of the economic objectives from all participants is necessary [8,9]. The flexibility implies new models in relation to new actors: Transmission System Operator (TSO), Balance Responsible Parties (BRP), DSO, and clients, including the FSP or aggregators. Thus, the organization of the new FS must guarantee the service payment and their management. At the distribution level, two main relation architectures have emerged in flexibility operations: a direct DSO-asset owner relation and a DSO-FSP, where the FSP can control different assets of different consumers.

There are different relationship scenarios amongst TSO, BRP, DSO, and clients, which make up different market models [10]. The markets allow one to perform tasks related to plan, bill, control, or settlement, but, in the European Regulation, the tasks and ambits of the involved parties are more strictly separated than those in the case of other non-European regions. Additionally, the degree of interaction between these parties depends on the market structure. One of the most widespread types of market organization is the Central Market including the day-ahead market and the intraday market, where OMIE (the Iberian market operator) is an example. However, there are some other emergent types of markets based on transactive energy, like distributed market with peer-to-peer communication. In this respect, various research groups and initiatives are proposing the deployment on energy markets of some new technologies, like Blockchain [11], providing secure, traceable, and reliable systems.

The solution proposed in this paper is the flexibility provided as a service, based on OpenADR standard protocol, which provides the system operator a demand-side management (DSM) tool, exemplified by a Capacity Bidding Program (CBP). Additionally, a Decentralized Permissioned Market Place (DPMP) is added to establish an incentive-based strategy. DPMP provided a Local Market in which the local aggregators and DSO can exchange (request, offer, and consume) FSs. In addition, a Global Market can be included in order to translate the FSs effects to the higher layers of the power grid. The DPMP provides a two-level Market Place in which the FS can be exchanged, and the DSOs have a public registration of the availability and reliability of FSP. The main contributions of this paper are:

- Highlight the usefulness of the OpenADR protocol as a DSM tool, showing the utility of its distributed architecture for managing and monitoring the consumption of energy assets.
- Proposal of FSs as a tool for grid management for DSOs by using the OpenADR protocol, providing an optimal use of energy resources and sustainable energy systems.
- Evaluation of a Local Market for managing the relationship between DSOs, FSP and asset owners.
- Implementation of a portable Virtual Top Node (VTN) at DSO level.
- Implementation of a Proof of Concept (PoC) and field tests at the Savona Campus (Savona, Italy).
- Successful application of the PoC in peak shaving scenarios.
- Design of DPMP to create an incentive model for the participation of FSs.

The description of the PoC developed and deployed in the University of Seville and Genoa University, coordinated by Enel, is described in this paper. In the first place, a brief explanation of OpenADR standard protocol is shown. In the second place, a bibliographic review about similar initiatives is included. In the third place, the project that framed the proposed solution is introduced. The developed and deployed solution is defined, the Local Market is described, and the picture of the solution is included from the high layer (with the DSO and CBP programs) to the low layer (physical devices). Finally, the experimental results and conclusions are included.

## 2. OpenADR Overview

**Figure 1:**
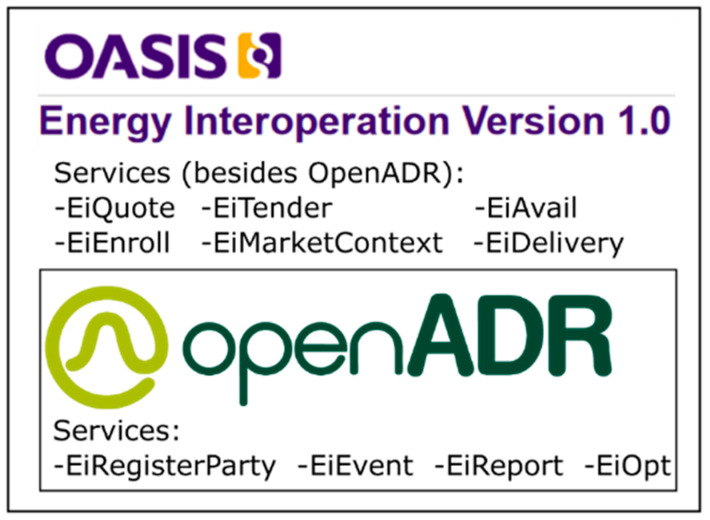
Open Automated Demand Response (OpenADR) Services vs. EI v1.0 Services.

The OpenADR is a protocol to allow Automated Demand Response (ADR) procedures, with the possible intervention of aggregators. OpenADR is an open standard and it is based on Energy Interoperation v1.0 (EI) from OASIS [12] (Organization for the Advancement of Structured Information Standards). The OpenADR 2.0b [13] implements some services specified in EI (see Figure 1). The main services implemented by OpenADR 2.0b are EiRegisterParty, EiEvent, EiReport, and EiOpt. These services allow the registration or modification of resources, events, reports, and options to participate in events, respectively. However, EI v1.0 implements all of them, adding other additional services, enabling implementation of a transactive energy approach.

According to its documentation, the OpenADR protocol is mainly oriented to the electric energy market (but it can also be used in the interchange of active power, reactive power, capacity, etc.). In this sense, the OpenADR protocol attempts to get an implementation of EI oriented to Demand Response (DR) management. There are several interfaces implemented which provide the functionality proposed by the OpenADR Standard. Notwithstanding, the University of Seville has developed a new implementation of a VTN with OpenADR protocol 2.0b, based on a modular structure and information standards. The VTN has been developed in order to have a good base to develop other protocols related to DR and DER, extending the OpenADR to cover the EI.

**Figure 2:**
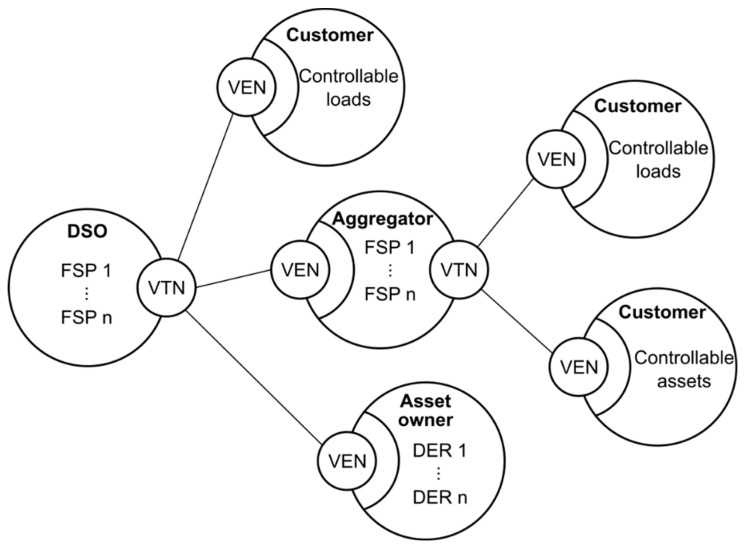
Hierarchical relation between Virtual Top Nodes (VTN) and Virtual End Nodes (VENs).

The architecture of OpenADR is based on interconnected nodes (see Figure 2), in which a VTN can communicate with one or more Virtual End Nodes (VEN). At the same time, the system in which VEN is deployed could act as an aggregator implementing a VTN in the lower layer. In this schema for the current version of OpenADR, the loops are avoided by constraints of the OpenADR protocol. In this way, a VEN could manage a system with a VTN that manages a Smart Building or a complete power grid, simplifying the downstream control and regulation of all the available resources. This concept is similar to the concept of Virtual Power Plants (VPPs) [14,15], which simplifies the management of the power grid. A VPP may play a role as a VEN and VTN (at the same time, like intermediate node in Figure 2) but, in this architecture, could include only final nodes with only one role (VEN, in case of leaf nodes, and VTN, in case of root nodes). Thus, this architecture allows more flexibility providing aggregator nodes and final nodes. The information exchanged is primarily based on control and notification functions. The report service provides information about different parameters from the nodes, according to the infrastructure behind each node.

Although this structure could be centralized, it is a distributed and hierarchical architecture similar to a tree structure, because a VEN could be in several hierarchical structures; this means that a VEN could take part in two or more structures with two different VTN as a root of the structure.

**Figure 3:**
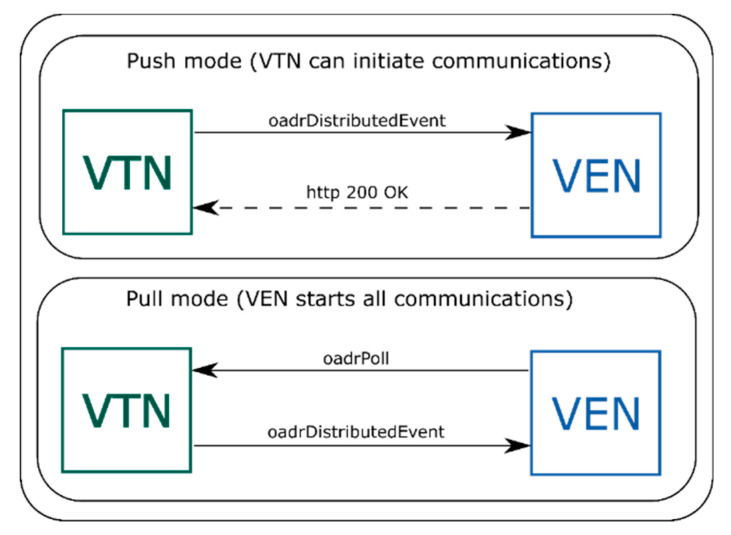
PUSH and PULL modes in OpenADR protocol.

Additionally, the OpenADR protocol can be based on Hypertext Transfer Protocol (HTTP) Representational State Transfer (REST) services or Extensible Messaging and Presence Protocol (XMPP) and Extensible Mark-up Language (XML) according to the standard. In the case of HTTP, the VTN supports both PUSH and PULL modes (Figure 3). This means that a VTN could have VENs in PUSH mode and in PULL mode at the same time, but one VEN cannot be in the two states at the same time, although it can switch between them. In PUSH mode, the VTN has an active role and it is able to start communications with the VEN. In PULL mode, the VTN has a passive mode, and it only responds to VEN requests. The VEN periodically polls the VTN in PULL mode to check if it has any pending tasks.

The OpenADR and EI standard are harmonized with the other standards from International Electrotechnical Commission (IEC). Additionally, there is a high grade of interoperability with other protocols like IEEE 2030 [16].

## 3. Literature Review

The OpenADR standard protocol has been used in different sectors of the energy business, with applications always related to ADR.

In the case of DER, a general case of feasibility and flexibility is provided by [8], establishing a clear methodology for VPP flexibility modelling, but involving TSO and DSO interfaces. Additionally, [17] described an application of the concept of VPP based on OpenADR standard protocol hierarchy for dynamic charging of automated guided vehicles. In this case, the VEN is implemented as a VPP, which is an aggregator of different DERs. Ref [18] describes the software components required for a generic scalable controlling entity ICT architecture, which does not require additional work for additional DERs and DER types, with communication negotiation based on IEC 61,850 and OpenADR.

In the case of DR, [19] developed incentive DR with a Commercial Energy Management System (CEMS) based on a diffusion model, smart meters and ECHONET Lite protocol implementing as a VEN inside an OpenADR-based infrastructure. An example of a communication interface between ECHONET and OpenADR is described in [20]. Ref [21] proposed an analysis of web service layer based on OpenADR to provide fast automated demand response (FastADR) services, four seconds is considered a reasonably FastADR communication time according to [13]. Ref [22] introduced a secure and interoperable DR management platform assisted with aggregators, involving multiple strategies and policies provided from energy market stakeholders, increasing the security by making use of Smart Contracts (SCs) and Decentralized Applications over OpenADR protocol. Ref [23] proposed a distributed demand management architecture to evaluate information exchange in the existing industry standard OpenADR.

In case of both functionalities, Ref [24] proposes a methodology based on an energy analysis of industrial processes to quantify and validate the flexibility potential of industrial customers to create a certification procedure, involving an infrastructure with aggregators, industrial customers, and energy service companies, among others.

There are other applications that deal with transactive energy concepts. Ref [25] presented a fog-based Internet of Energy (IoE) architecture for transactive energy management systems, designed with three different layers, supporting different communication protocols. Ref [26] proposed an IoT (Internet of Things) system for the accounting of energy flows, as well as a blockchain approach to overcome the need for a central control entity, allowing the creation of local energy markets.

There are other points of view; Ref [27] provides a standardized framework, in which the OpenADR protocol is included, to remove a significant barrier for enabling farms (agricultural) to provide services to the electricity grid while improving their balance, defining some concepts: the current and future needs of the electricity grid, the available electricity market mechanisms through which farms can provide services to the grid, and understanding the prosumer equipment of farms. Thus, the farmers explored sustainable irrigation water and energy management practices. The solution proposed in the current paper provides a tool to manage congestion provoked by prosumers with the management coordinated by DSOs. Additionally, the solution proposed in the current paper could be implemented in different regulations.

The development of decentralized markets is described in [28], providing a socio-technical evolution of the application of decentralized technologies in the energy sector, and [29] made a systematic review of challenges and opportunities of blockchain technology in the energy sector.

In the case of decentralized markets based on Blockchain, Ref [30] introduced a decentralized market design, which allows a DSO to manage local demand constraints by obtaining flexibility from competing aggregators, which must in turn incentivize prosumers to provide this flexibility, the authors simulated a proposed market design using the IEEE European Low Voltage Test Feeder. [31] defined an optimization procedure to generate a solution pool with diverse schedules for the coordinating approaches, developing two novel coordinating decentralized optimization approaches: Parallel Successive Cluster Optimization (PSCO) and Parallel Successive Cluster Optimization with Iterative Desync Algorithm (PSCO-IDA). Ref [32] presented a review and classification of existing DER as flexibility providers and a breakdown of trading platforms for DER flexibility in electricity markets. The solution proposed in the current paper provides a tool to manage congestion based on an open standard and on an open technology. On one hand, the OpenADR standard allows management of congestion in a hierarchical way, and, on the other hand, the DPMP provides a tool to manage the incentives and the reliability of FSPs. Both technologies could be implemented in different regulations and is based on open and tested technologies.

For DSO integration scenarios with service providers such as aggregators, several proposals were found. Ref [33,34] proposed DSO platforms to manage flexibility resources through aggregators, and simulated results are obtained in both references. In [35], a DSO platform to enable smart prosumers (at building level) to participate in DR programs is presented; the platform was part of the H2020 project DRIMPAC and is also based on OpenADR, but no results of the platform have been published yet.

The solution proposed in this paper contributes with a portable tool available for DSOs to manage FSs based on OpenADR Standard and Local Markets (with Decentralized Permissioned Local Markets), and, in addition, developing a PoC with real FSP supported by the Enel DSO company. To the best of our knowledge, no similar results with the scale of the proposed PoC for a DSO-aggregator have been published yet.

## 4. The Grid Flexibility & Resilience Project

**Figure 4:**
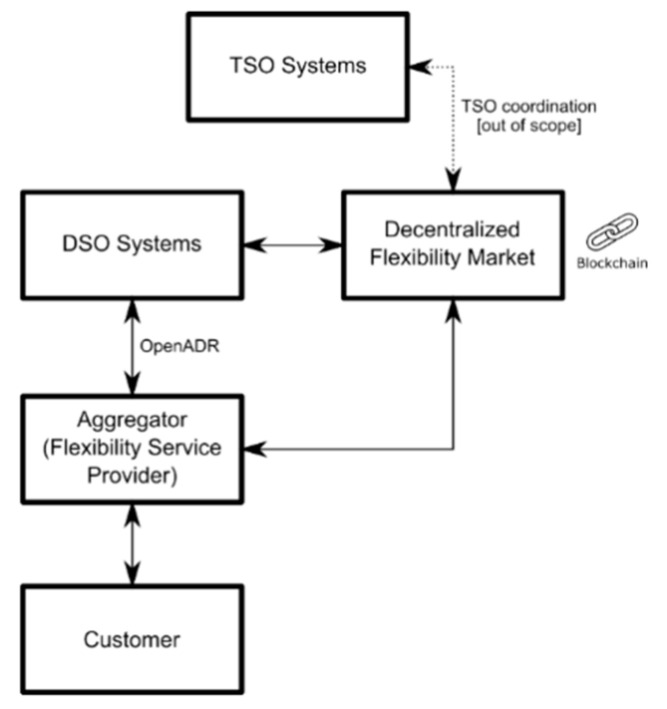
Hierarchy of the Proof of Concept (PoC). The PoC is the result of a successful collaboration between Enterprise and University.

The proposed solution was developed in the context of the Grid Flexibility & Resilience Project. The general objectives and results during its first phase are described in conference paper [36], while this paper is centered on the results of the second phase. This paper provides the details of the technology used in the PoC based on flexibility as a service and an aggregator with an intelligent system to manage Aggregated Energy Infrastructure, integrating DERs. The aggregator is controlling an infrastructure located in Savona (Italy) with the collaboration of Enel and Genoa University. The aggregator is implemented by ROSE [37], a system developed by MAPS group. Thus, the objective of this phase is to test OpenADR (Open Automated Demand Response) protocol, according to the OpenADR Alliance guidelines, by sending OpenADR 2.0b signals from a basic Demand Response Management System (DRMS) to an infrastructure controlled by an aggregator. Figure 4 shows the conceptual graph, establishing the scope of the project.

## 5. Local Market

**Figure 5:**
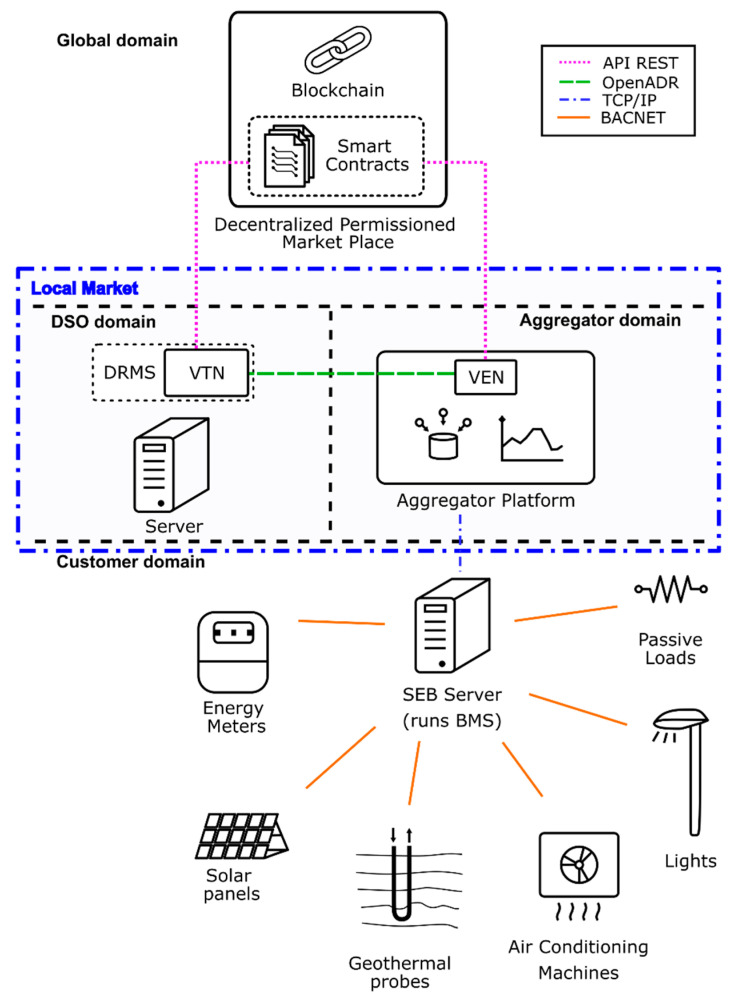
Scheme of PoC.

The developed and deployed infrastructure as a result of the proposed initiative is shown in Figure 5. The VTN is used to send events to the VEN. The VEN at the Smart Energy Building (SEB) located at the Savona Campus server is remotely controlled by the aggregator platform. The SEB is based on Building Automation and Control Networks (BACnet) devices. Initially, the events are generated by a local system because the system is not connected to the DSO infrastructure. The VEN is connected to an aggregation platform, that decides which of the available resources must be used in the DR program and sends them control orders to fulfill the DR event requirements. The connection between systems is under a VPN in order to keep the channel secure.

The Local Market offers a place to exchange FS in which the expected actors are the DSO, the aggregators, the retailers, or some other ones, which could have access to DERs providing FSs to the DSO or other aggregators in the same grid zone. This Local Market is separated from the Global Market because the Global Market trades with energy, not with flexibility.

The Local Market is based on application of OpenADR with incentive management. The incentive management is based on a DPMP, the incentive shows the confidence in the aggregator.

**Figure 6:**
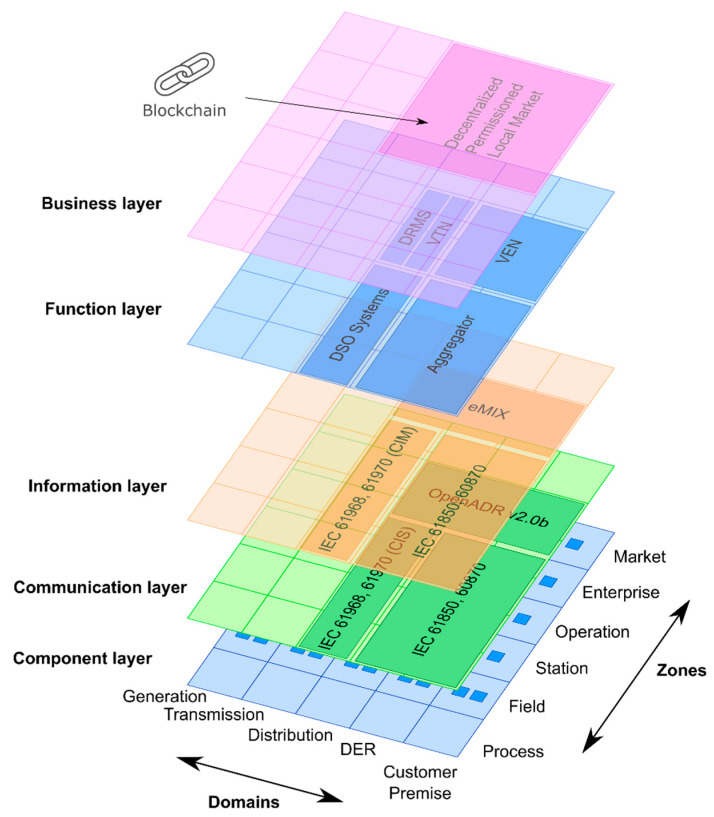
Architecture according to Smart Grid Architecture Model (SGAM).

The integration of Local Market based on OpenADR and DPMP is shown in Figure 6. The integration is shown according to SGAM (Smart Grid Architecture Model) [38,39,40], the proposed model tries to provide a specification of implementation of the proposed architecture coordinated with a DPMP.

### 5.1. Security Issues

The security issues are tackled thanks to several conditions adopted in the PoC:

- The usage of Virtual Private Network (VPN).
- The message payloads include Standard Security levels with Transport Layer Security (TLS), but it is possible to increase the security level including XML signatures combining with RSA and Elliptic Curve Cryptography (ECC).
- The VEN needs to be registered in the VTN.
- The payload message includes several identifiers (depending on the message). The requests and responses must know the sequence of identifiers exchanged between VEN and VTN.

The proposed system allows exchanging information in a controlled environment in both modes: PULL and PUSH.

### 5.2. Decentralized Permissioned Market Place (DPMP)

**Figure 7:**
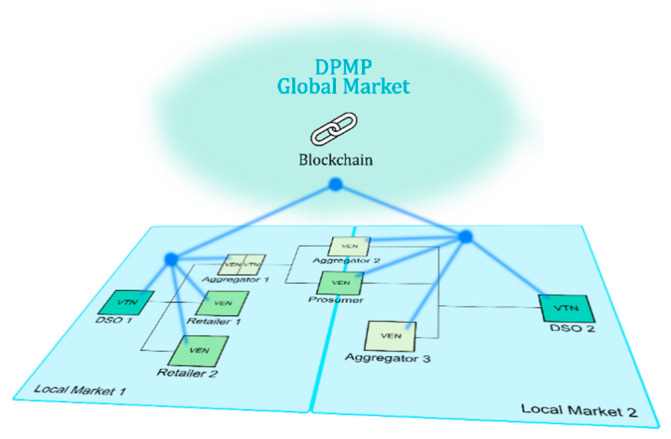
Decentralized Permissioned Market Place (DPMP) and Local Markets integration schema.

DPMP is a global market in charge of Flexibility Incentive Management (FIM). The quantity of incentive shows the confidence, resilience, and efficiency of FS offered by an aggregator or other type of actor. Although the FS are offered in a Local Market, the FIM is made in a global market (Figure 7). In DPMP, aggregators and other actors could exchange incentives awarded in any Local Market. Additionally, the aggregators could be awarded or penalized because of the participation in the flexibility in each Local Market. The aggregators are penalized in case of non-compliance of service agreements. Thus, DPMP shows a global view of flexibility in the grid, and the aggregators and other actors could have a tool to globally manage their incentives. The incentive management and the interaction with the VEN and VTN is based on the simultaneous calling to a SCs in a permissioned Blockchain [41]. The Blockchain technology is based on Quorum. The core of Quorum is Ethereum (a public blockchain) with an additional permissioned layer, controlling the access of different clients.

The volume of incentives in an aggregator provides an additional indicator for clients of DPMP and provides an opportunity for clients to offer better resources to their clients. The incentives are awarded according to the reduction offer. Thus, when the DSO requests FSs to the aggregator, they are awarded or penalized according to the offer. The FSs are geo-referenced according to the topology of power grid, but the incentives are independent and could be exchanged between aggregators. The incentive is not tested in this case, because it is out of the scope of this paper.

Additionally, the DPMP provides services related to the traceability of incentives, clients, etc. Thus, the traceability provides an additional parameter to evaluate the client in all the Local Markets in which the client is involved.

The main vulnerability of this architecture is the consensus algorithm. Currently, the consensus algorithm is based on Raft [42] and Istanbul Byzantine Fault Tolerant (IBFT) [43]. It is necessary to define a new consensus algorithm which could include additional factors to provide a better and secure consensus method, for example, including topological grid constraints, which are not considered in the two mentioned examples. The inclusion of implicit location information will provide additional advantages to apply a consensus algorithm quicker, increasing the number of transactions and reducing the computing requirements for the consensus process. For example, supposing an FSP which is participating in two Local Markets in different locations in the power grid, in one of the Local Markets the FSP could show a high availability and reliability to provide FS, getting a high rate of incentives. However, in the other Local Market, this FSP is not a good FSP, getting penalties. Thus, using the Global Market or a sidechain, the FSP could exchange incentives between different Local Markets to maintain the reliability level. The inclusion of location as an additional factor could provide a threshold of FSB reliability. This means that incentive is not only an incentive like money, this incentive generates trustworthiness for the FSP.

The interaction between clients and DPMP is performed by means of SCs:

- createEventSC. This is a SC available for VTNs. This SC creates an event or flexibility scenario, which has associated information about flexibility scenario: flexibility characteristics, the Local Market in which the FS is offered, and an incentive proposal. This SC is also used to update the information of the Event. If the Event identifier is modified, it will be necessary to remove the Event and create it again. The createEventSC could consume offers previously added by addOfferSC or addTransactiveOfferSC, or could provide a flexibility scenario, and the VENs (aggregators, etc.) could involve using optInSC.
- optInSC. This is an SC available for VENs and VTNs. This SC updates the information associated to a flexibility scenario, involving resources in one flexibility event from an aggregator (partially or totally).
- optOutSC. This is an SC available for VENs and VTNs. This SC updates the information associated to a flexibility scenario, taking off resources from an aggregator (partially or totally).
- addOfferSC. This is an SC available for VENs. This SC creates a flexibility offer related to a VEN with a Resource. The Resources have a location associated to determine the availability for different aggregators. This SC is also used to update the information of Offer.
- removeOfferSC. This is an SC available for VENs. This SC removes a flexibility offer created by addOfferSC. There are some scenarios: the offer is just accepted by an aggregator, in this case, the VENs cannot remove the offer, the VENs have to invoke the optOutSC and it will be penalized; the other scenario is when the offer is not accepted by any aggregator, in this case, the offer is removed and the next offer will need more gas to be processed.
- addTransactiveOffersSC. This is an SC available for VENs and VTNs. The aggregator can offer a FS due to a previous reservation of other offers that provide flexibility in its zone. These offers are characterized by the previous offers and are constrained by these offers. A transactive offer could be created using other transactive offers. This SC is also used to update the information of Offer.
- removeTransactiveOffersSC. This is an SC available for VENs and VTNs. The aggregator (VEN or VTN) can remove a flexibility offer based on other offers. This case also has two scenarios, like removeOfferSC. If an original offer (root of transactive offers) is removed, a whole chain of offers and transactive offers are automatically removed or opted-out.
- traceabilitySC. This is an SC available for VENs and VTNs to check the validity of transactive offers and offers of FSs. This SC checks the whole chain to check all offers involved in the request, offering a trusted and secured verification of resources and offers. This SC provides information about power load balance.

Additionally, this market could have other clients, like TSOs, to include balance constraints, although this type of SCs is still under research.

### 5.3. Capacity Bidding Program (CBP)

A Capacity Bidding Program (CBP) is a DR program that offers participants various flexibility product options by which they can earn incentive payments in exchange for reducing energy consumption when requested by the Utility. This program is available for big customers (e.g., industrial customers), and for aggregators.

In a CBP, customers offer a certain amount of capacity of reduction of its consumption for a certain amount of time. Offers must be sent to the aggregator or to the DSO (depending on the regulation) and could be recalculated/changed every day, every month or they can be fixed.

The proposed CBP is based on the programs applied by the companies San Diego Gas & Electric (SDG&E) [44] and Pacific Gas & Electric (PG&E) [45]. Although these examples are oriented to residential consumers, the different cases show the fundamentals and the viability of different technologies.

Specifically, a utility-aggregator structure is deployed to apply this program as a PoC. This structure is made up of three elements: a DRMS in the University of Seville (acting as the DSO), an “aggregator”, and the asset owner in the Savona Campus (Genoa University).

A CBP requires a bidirectional communication between customers (or aggregators) and the Utility. The OpenADR standard protocol is used as a communication method, as it includes a set of signals/events to implement DR programs, so it could be used to implement communication channels and establish the methods and format of information flow between the Utility (as VTN) and a customer/aggregator (as VEN).

This architecture implies the installation of a VTN in Seville and a VEN in Savona Campus, and specifies what information must be exchanged between them.

### 5.4. VTN Platform in University of Seville

In this project, the VTN is placed at the University of Seville, while the VEN platform is located on Savona Campus in Genoa University, being the aggregator Platform developed and deployed by MAPS Company. The communications are performed under a Virtual Private Network (VPN), requiring the manual pre-registration of VENs in the VTN. Therefore, the VTN only performs actions with pre-registered VENs and ignores the others. Additionally, the VTN address is pre-configured in VENs which will partake in the process, avoiding the “man-in-the-middle” attack.

**Figure 8:**
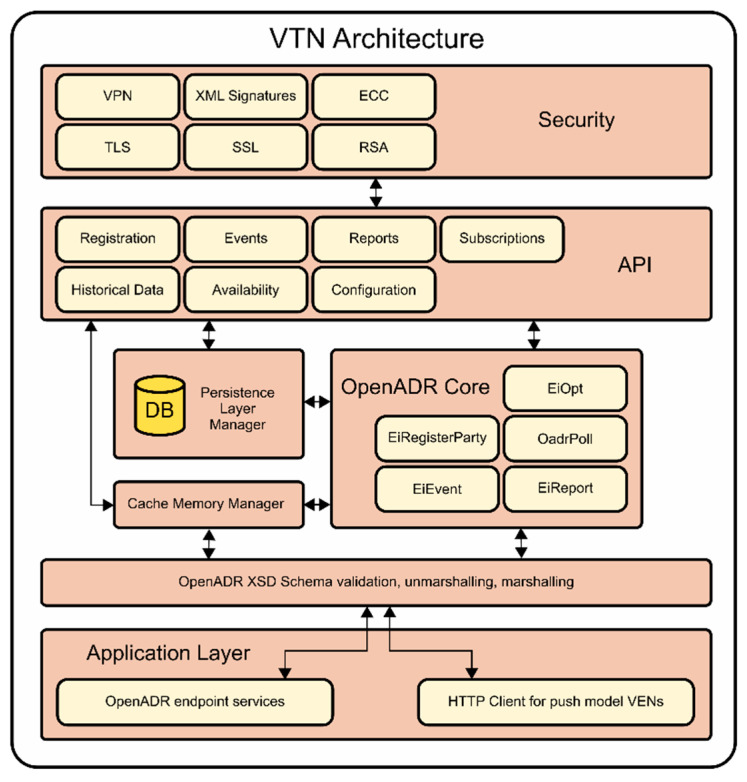
VTN Architecture.

Although, the proposed system is not still connected with the DSO, the DSO operations were simulated. The main actor of the DSO is the DRMS which uses the information manually loaded, providing a new scope of services and more flexibility in DR operations. The proposed system was developed modularly to provide a Demand Response Automated Server (DRAS). Thus, the VTN and the DRMS are independent modules. The VTN shown in Figure 8 provides different APIs, some of them are oriented to serve to VENs and others to serve higher systems like DRMS. The VTN has a core, in which the main services are implemented (EiRegisterParty, EiReport, EiEvent, EiOpt, OadrPoll) supporting polling and pushing services. Additionally, the VTN keeps its own configuration in a cache memory. If any fault or error occurs, the system could get the most recent information from the Persistence Layer Manager, a physical copy of the previous state of the VTN. The VTN is completely written in Java.

The Cache Memory Manager and the Persistence Layer Manager make the VTN faster and more reliable, with high tolerance in case of node fault.

The DRMS is directly connected to the VTN, providing an administration interface where all the operations and services related to the registered VENs are available to be used.

### 5.5. Savona Campus

**Figure 9:**
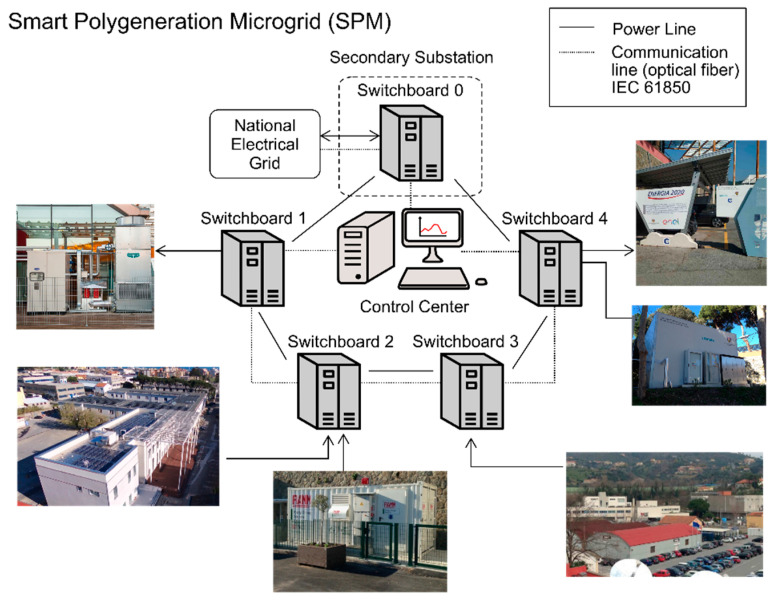
Smart Polygeneration Microgrid (SPM).

The role of customer corresponds to the Savona Campus (Genoa University) organized as a Smart Polygeneration Microgrid (SPM) (see Figure 9): a three-phase low voltage (400 V line-to-line) “intelligent” distribution system running inside the Campus and managing energy resources:

- 2 micro-cogeneration gas turbines (µGT) fed by natural gas (rated power: 65 kWel and 112 kWth each);
- 2 photovoltaic fields (PV) (95 kWp);
- 2 absorption chillers (AC) employed to refrigerate two buildings during the summer;
- 1 electrical storage systems (ES) (Na-NiCl2, 140 kWhel);
- 2 standard electrical vehicle (EV) charging stations;
- 2 Vehicle to Grid (V2G) charging stations; and
- a 500 kWth gas boiler

**Figure 10:**
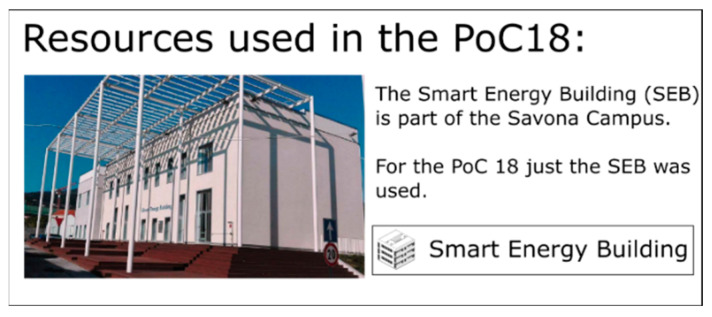
Smart Energy Building (SEB) in Savona Campus (Genoa University, Seville).

The SEB, shown in Figure 10, is equipped with a BMS, a geothermal heat pump (45 kWth) and a PV field (23 kWp) that are directly connected to the microgrid. The geothermal heat pump installed on the SEB typically absorbs up to 15 kW and can be remotely controlled via BACnet. This allowed this asset to be used as a controllable load in the context of the PoC.

**Figure 11:**
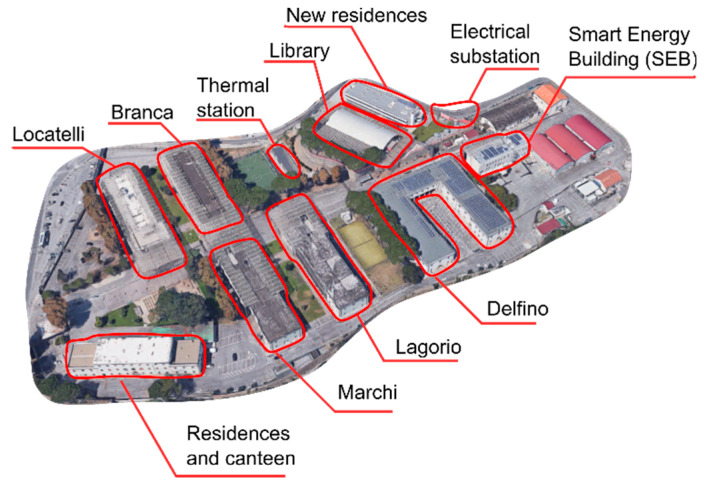
Savona Campus buildings (Genoa University, Italy).

The microgrid is also connected to the pre-existing Campus network feeding the Campus buildings, shown in Figure 11.

## 6. Experimental Results

**Figure 12:**
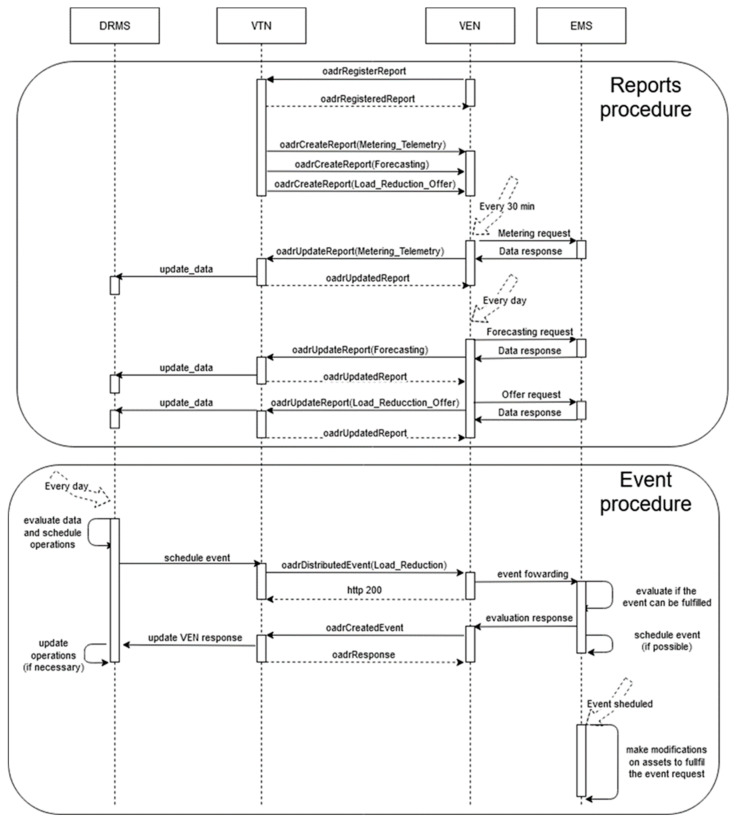
Sequence diagram of communication between VTN and VEN.

The real operation tests of the OpenADR protocol were performed in December 2018. Four of them are shown here, occurring between the 10th day to the 14th. Although there were three weeks of tests, this period was finally selected because of the weather conditions, in which the use of Heating, Ventilation, and Air Conditioning (HVAC) systems is necessary. Additionally, the DERs used in the PoC have some requirements for its normal use (University Institution) and they can only be used in some periods of the year in order to keep the normal operation of the Savona Campus. The summary of the executed real tests is summarized in Table 1. The reduction of consumption is about 50% in each event, reaching 74.4% on the last day. The operations necessary to perform the consumption reduction is oversaw by the aggregator. But, unfortunately, the OpenADR conformance rules do not specify anything about the aggregators. If they are involved in an event and when the event is fired, none of them do not carry out any action to reduce consumption, and the aggregator is penalized in DPMP. The aggregator should send an “optOut” by means of EiEvent or EiOpt services before the event begins in order to give up the event constraints and avoid the penalization in DPMP. Figure 12 shows the sequence diagram of communications between VTN and VEN in one of the performed tests (all tests have the same structure); the period between messages is the parameter which would be different between different tests. In the Reports procedure (Figure 12), the reports include data from VENs, according to the information available. This information is periodically updated and provides the offers for the DPMP (addOfferSC and removeOfferSC). In the Event procedure, the oadrDistributedEvent establishes the initial action for configuration of the flexibility scenario; the aggregators may or may not be involved in this flexibility scenario, providing FS. This way, the DPMP is proposed to view the global confidence of aggregators. In these cases, in the Event procedure (Figure 12), the oadrDistributedEvent and oadrCreatedEvent provokes a call to the corresponding SCs: createEventSC and optInSC. These increase the incentives available for the aggregator in the DPMP. When the event has finished, the DRMS checks whether the event has been performed by aggregators. In the case that an aggregator has not participated in the event, the DRMS through VTN updates the state of the aggregator (opt out option) in the Event procedure, calling optOutSC in the DPMP. In the proposed tests, the offers are provided by VENs in the previous day using the report services. Additionally, the information is updated in the DPMP by using addOfferSC or addTransactiveOfferSC. The VTN chooses among these offers using the event services and the createEventSC in the DPMP.

The information sequence between the VTN and VEN starts with the registration process. In this process, the VTN requests the information available in the VEN, and the VEN uses the Report service to periodically send information from the aggregator.

The trigger date and the details of the consumption reduction are set by requesting an event from the VTN to the VENs. The VEN and the aggregator manage the downstream power grid, satisfying the established restrictions. The VTN is independent from the control of the downstream resources from the VEN, and the VEN is independent from the control and management made upstream from the VTN.

**Figure 13:**
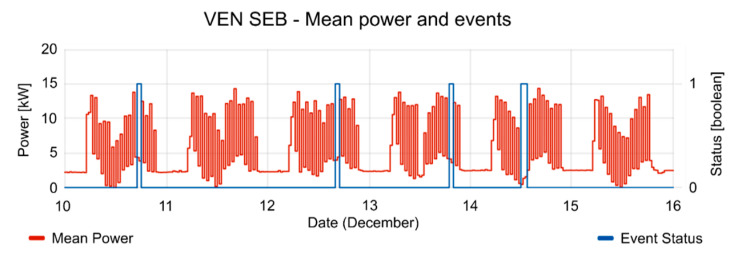
Mean power and event status (where 1 is active and 0 is inactive) during the test period.

**Figure 14:**
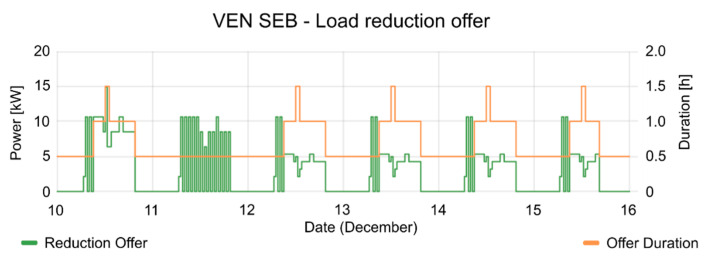
Load Reduction and duration of each offer during the testing period.

**Figure 15:**
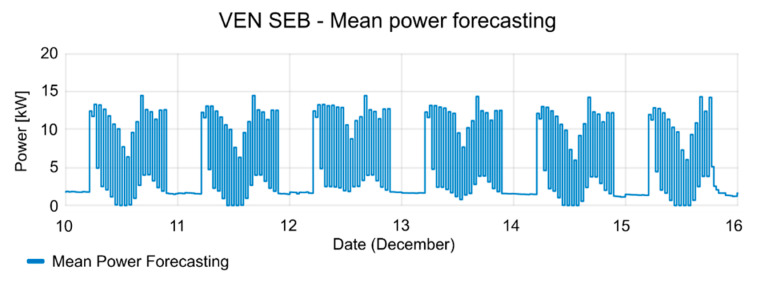
Forecasting of consumption during testing period.

The consumption of the SEB had strong fluctuations during the test periods. The mean power of SEB and the active period of events are shown in Figure 13. In the active period of events, the consumption of the SEB is reduced, according to the reduction offer; offer duration (see Figure 14), and consumption forecasting (see Figure 15), which were previously provided by the VEN.

**Figure 16:**
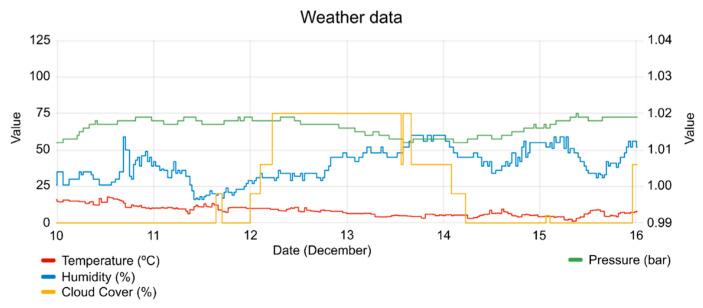
Temperature, humidity, pressure and cloudy during the test period.

The DRMS considers other parameters, like temperature, humidity, pressure and cloudiness (see Figure 16), trying to adjust the load dispatch events when the SEB could reduce without giving up the well-being of the buildings’ occupants. These parameters have a high influence on solar energy [46].

**Figure 17:**
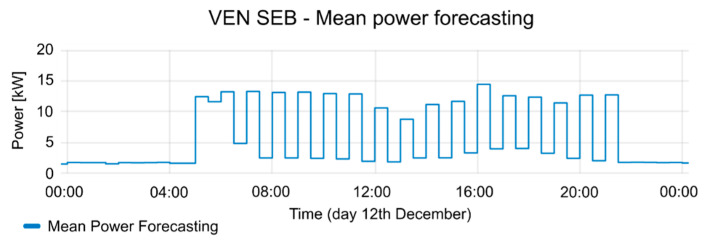
Consumption forecasting for 12 December test.

**Figure 18:**
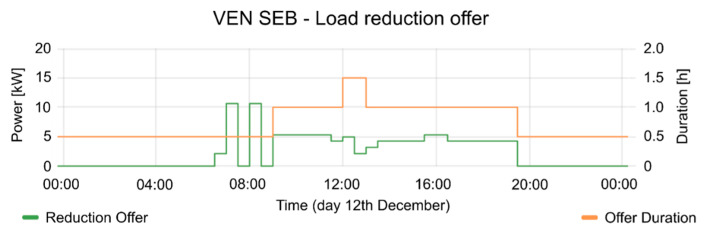
Offers of reduction and their duration for 12 December test.

**Figure 19:**
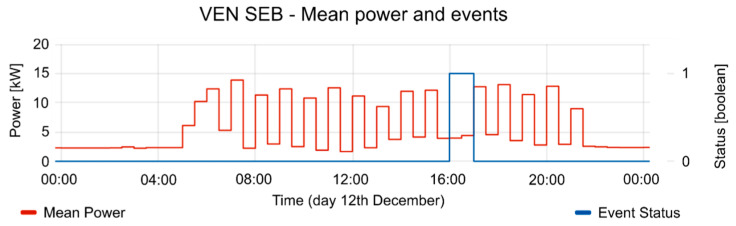
Mean Power and event status (where 1 is active and 0 is inactive) for 12 December test.

Figure 17, Figure 18 and Figure 19 contain the results of tests performed on 12 December. The consumption forecasting is around 10 kW and is lower between 12:00 and 14:00 (see Figure 17). The reduction offer was near 5 kW (see Figure 18). The offer duration rises between 12:00 and 13:00, while the mean duration of the offers is 1 h. Thus, the DRMS proposes a reduction between 12:00 to 13:30 (see Figure 19), taking advantage of the maximum reduction of consumption. The VTN only requests an event with the data provided by the different VENs. The VEN performs the reduction, complying the requirements from VTN. Once the event is triggered, the consumption decreases according to the constraints established by the VTN event. This guarantees the correct operation of the power grid. The event in the OpenADR protocol allows one to define a ramp up period, in which the VEN platform takes the necessary actions to carry on with the event constraints, performing the consumption reduction.

**Figure 20:**
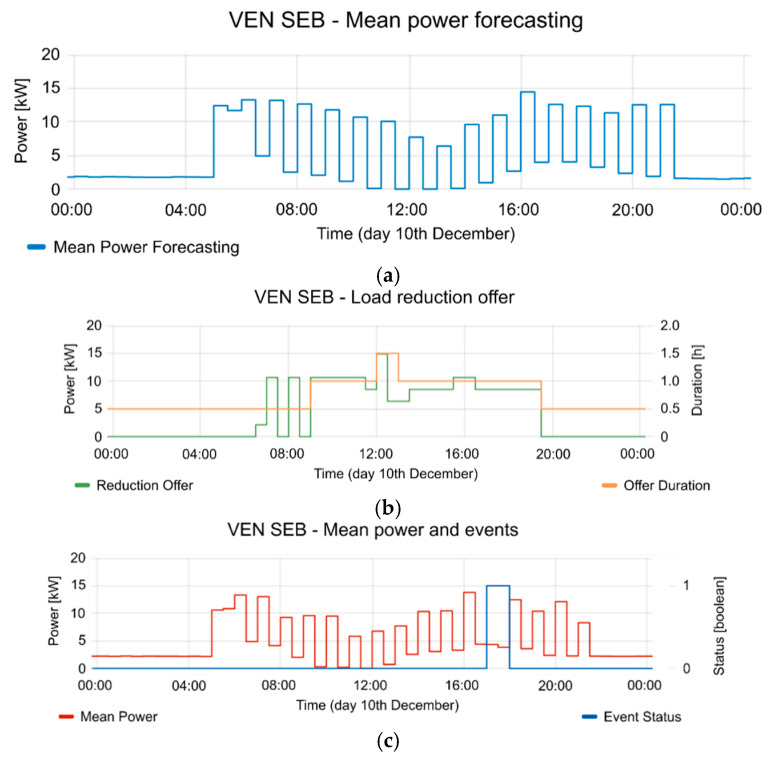
Detailed information from 10 December event. (**a**) mean power forecasting; (**b**) reduction offer and offer duration; (**c**) mean power and event status (where 1 is active and 0 is inactive).

**Figure 21:**
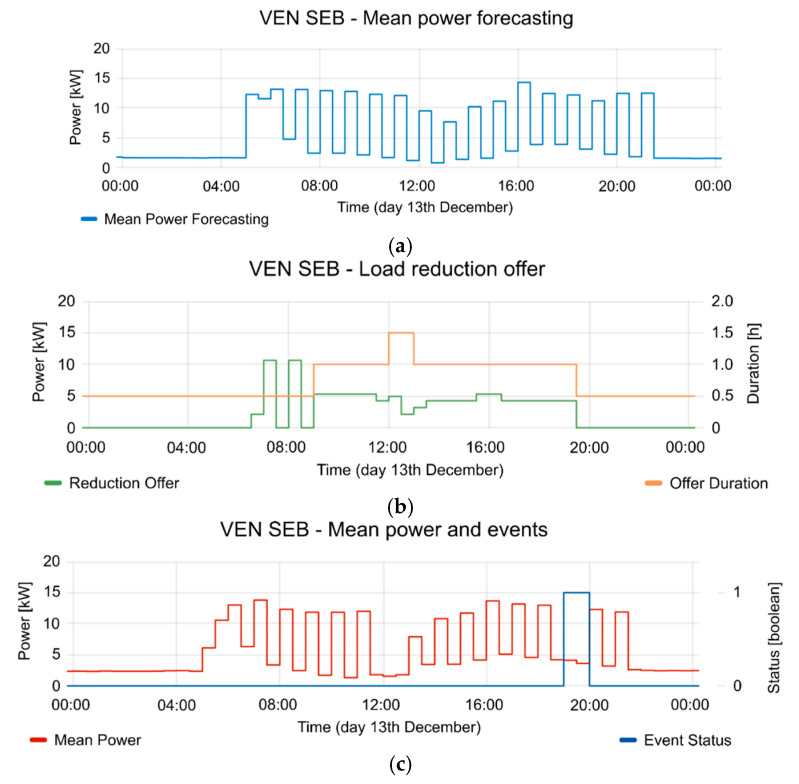
Detailed information from 13 December event. (**a**) mean power forecasting; (**b**) reduction offer and offer duration; (**c**) mean power and event status (where 1 is active and 0 is inactive).

**Figure 22:**
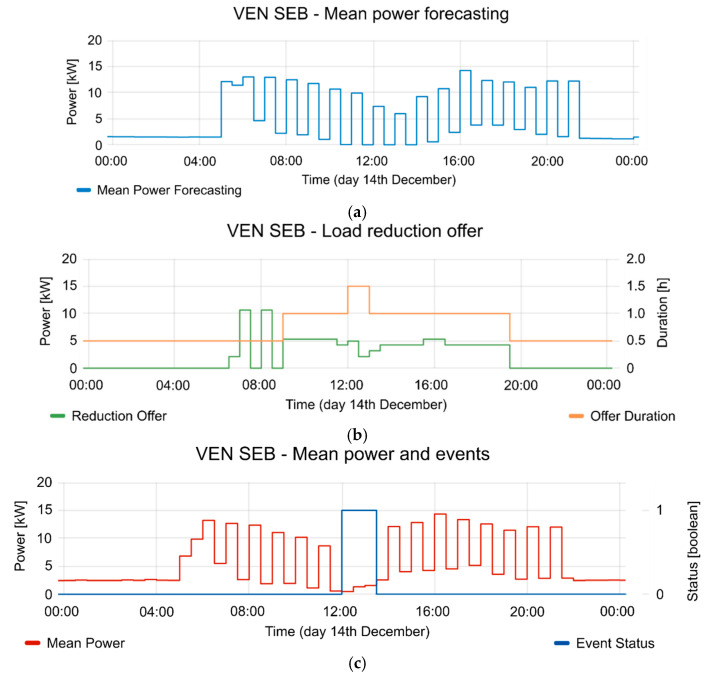
Detailed information from 14 December event. (**a**) mean power forecasting; (**b**) reduction offer and offer duration; (**c**) mean power and event status (where 1 is active and 0 is inactive).

Figure 20, Figure 21 and Figure 22 show the detailed view of 10, 13 and 14 December, respectively. In each figure, the mean power forecasting is shown (“a” section of each figure) for each day. Forecasting is provided by the aggregator. The reduction offers and offer duration is shown in section “b” of each figure. The mean power and event status are shown in section “c” of each figure. The event status is 1 in case the event is active and 0 in case the event is inactive. As can be seen, the best reduction was obtained on 14 December, but the other days also show the advantages of implementing a CBP. For example, 12 and 10 December show a similar reduction range, although 13 December shows a reduction of 42.4%, however it is necessary to consider the time period involved in the event. Additionally, the reductions were applied in different periods of the day for each test. This fact plays an important role in the FS, because these periods are gathered from information provided by VEN in the report messages. Of course, the presented case could be extended to other types of FSs in the future. In all the tests, the aggregator responds to the flexibility request. This flexibility request has been designed in the VTN according to the offers provided by the aggregator. The VTN creates an event to take advantage from the FSs. When the event is in active period, the aggregator reduces the consumption according to the event constraints and requests. The proposed scenario is a sample of the advantages that could provide the technology, in a Local Market with several aggregators. Each aggregator could provide different offers and the DSO could take advantage from a variety of them with only one event. In this way, the congestion management could be performed with only one event, based on offers and demand-respond forecasting. Additionally, the VTN may manage different resources with different events because it is possible to limit the event for some specific resources.

## 7. Conclusions

The successful collaboration between Universities and Enterprises (each entity playing a different role in the system) is one of the most outstanding issues in this project, and they have been demonstrated in the results. The described results provide a reasonable vision of the potential of the OpenADR protocol. The usage of this protocol and algorithms for forecasting consumption and DR management provides a powerful tool to implement, not only CBP, but FS platforms to manage the capacity and congestion, increasing the stability, reliability, quality, and, therefore, making possible a reduction in the consumption. The test of this protocol in the context of DSO level provides the scenario to define a Local Market, in which the DSO could offer FSs by the interaction with TSO or consume FSs provided by the aggregators. Additionally, the compatibility of OpenADR, IEC and EI standards guarantees a long-life period, the interoperation with other standards, and a possible evolution to a transactive energy strategy [47].

Additionally, the designed DPMP provides a global tool to FIM, and a useful indicator of the confidence and efficiency of aggregators and DSOs. Therefore, the proposed solution provides a global market to evaluate the flexibility in different Local Markets, and the proposed Local Market could be implemented in any scenario, as it is based on standard protocols and it is implemented modularly.

The proposed solution provides a FSs management platform by using open standards and technologies, OpenADR standard and blockchain, respectively. The novel application of the standard protocol in the congestion management brings an important advantage. With this platform, the DSO could implement a FS management for congestion management while complying with the European regulations. In other countries, like the North American or Latin American market, this is not a regulation constraint.

The proposed platform is designed to provide a Local Market to manage the FSs incentives at DSO level. The Local Market is a channel to register and report FSs between FSP and DSO, the incentives are the representation of the offering, requesting, and accomplishment of FSs.

The implementation of portable VTN is independent from platform, thus it could be used to manage the flexibility or a DR program that includes the usage of FSs.

Additionally, the implementation of PoC based on a specific use case, like a CBP. CBP defines the different applications deployed in the hierarchical structure based on VTN and VEN, taking part in a real scenario, with an aggregator with a real SCADA, controlling real resources.

The authors did not find any similar results in any published reference with the scale of the proposed PoC for a DSO-aggregator, and the successful application of the PoC in the peak control scenario.

Although the DPMP was not tested with a complete scenario with several Local Markets and Global Markets, the authors propose a Global Market for FIM based on DPMP, providing a useful tool for Local Markets to manage the reliability of FSP on the Local Markets.

Finally, the main limitations of the proposed approach agreed on the future lines research derived from this project:

- The evolution of proposed system to be available for transactive energy environments, adding the rest of functionalities described by EI [12].
- The testing of synchronization of different Local Markets, with a global market.
- Testing of different incentive policies, compatible amongst different Local Markets and with global markets.
- Researching on new products and services and innovative business models.
- The simulation of DPMP with different Local Markets, involving several DSOs and aggregators (involving several Local Markets).
- Researching of new consensus algorithm with geo-referenced constraints.
- The participation of TSO in the global market, implemented by TSO.

## Figures and Tables

**Table 1:** Results of the real test performed with the proposed platform. Four days were selected.

Trigger Date (2018)	Trigger Hour	Duration (min)	Offer (Wh)	Forecasted (Wh)	Real Consumption (Wh)	Reduction (Wh)	Reduction (%)
10 December	17:00	60	8510	8330	4100	4230	50.8
12 December	16:00	60	5320	9209	4190	5019	54.5
13 December	19:00	60	4260	6720	3870	2850	42.4
14 December	12:00	90	4970	8880	2275	6605	74.4

## Abbreviations

**Abbreviation**	**Meaning**
ADR	Automated Demand Response
BRP	Balance Responsible Parties
CBP	Capacity Bidding Program
CEMS	Commercial Energy Management System
DER	Distributed Energy Resources
DPMP	Decentralized Permissioned Market Place
DR	Demand Response
DRAS	Demand Response Automated Server
DRMS	Demand Response Management System
DSM	Demand-Side Management
DSO	Distribution System Operator
ECC	Elliptic Curve Cryptography
EI	Energy Interoperation
FastADR	Fast Automated Demand Response
FIM	Flexibility Incentive Management
FS	Flexibility Service
FSP	Flexibility Service Provider
HTTP	Hypertext Transfer Protocol
HVAC	Heating, Ventilation, and Air Conditioning
IBFT	Istanbul Byzantine Fault Tolerant
IEC	International Electrotechnical Commission
IED	Intelligent Electronic Devices
IEEE	Institute of Electrical and Electronics Engineers
IoT	Internet of Things
OASIS	Organization for the Advancement of Structured Information Standards
OpenADR	Open Automated Demand Response
PG&E	Pacific Gas & Electric
PoC	Proof of Concept
PSCO	Parallel Successive Cluster Optimization
PSCO-IDA	Parallel Successive Cluster Optimization with Iterative Desync Algorithm
REST	Representational State Transfer
SC	Smart Contract
SCADA	Supervisory Control and Data Acquisition
SDG&E	San Diego Gas & Electric
SEB	Smart Energy Building
SPM	Smart Polygeneration Microgrid
TLS	Transport Layer Security
TSO	Transmission System Operator
VEN	Virtual End Node
Volt-VAR	Voltage and Reactive Power
VPN	Virtual Private Network
VPP	Virtual Power Plant
VTN	Virtual Top Node
XML	Extensible Mark-up Language
XMPP	Extensible Messaging and Presence Protocol
